# Transstadial and transovarial perpetuations of *Borrelia venezuelensis* in *Ornithodoros rudis*, with demonstration of vector competence

**DOI:** 10.1007/s10493-026-01124-z

**Published:** 2026-04-08

**Authors:** Felipe R. Jorge, Glauber M. B. de Oliveira, Igor S. Silito, Matheus Pasini Martins, Sebastián Muñoz-Leal, Marcelo B. Labruna

**Affiliations:** 1 Department of Preventive Veterinary Medicine and Animal Health, Faculty of Veterinary Medicine and Animal Science, University of São Paulo, Av. Orlando Marques de Paiva, 87, Cidade Universitária, São Paulo, 05508-270 SP Brazil; 2 Department of Animal Science, Faculty of Veterinary Sciences, Universidad de Concepción, Av. Vicente Méndez 595, casilla 537, Chillán, Ñuble, Chile

**Keywords:** Relapsing fever, Borreliosis, Argasids, Soft ticks, Brazil

## Abstract

Ticks of the genus *Ornithodoros* are recognized vectors of relapsing fever group *Borrelia*, such as *Borrelia venezuelensis*, whose infection dynamics in its vector, *Ornithodoros rudis*, remains poorly understood in Brazil. This study aimed to investigate the transstadial perpetuation and transovarial transmission of *B. venezuelensis* in *O. rudis* and the vector competence of all parasitic stages of this tick species. Experimental colonies were maintained under controlled laboratory conditions, and ticks were fed on Syrian hamsters (*Mesocricetus auratus*). Infection in hamsters was monitored using dark field microscopy and real-time PCR. *Borrelia venezuelensis* was detected across multiple tick life stages, and its presence in larvae derived from infected females confirmed transovarial transmission. Vector competence for *B. venezuelensis* was demonstrated for larvae, nymphs and adults of *O. rudis.* Additionally, neurological symptoms and sudden death were observed in some hamsters, possibly associated with toxicosis due to heavy tick infestation. These findings provide the first experimental evidence of transstadial and transovarial maintenance of *B. venezuelensis* in *O. rudis*, reinforcing its role as a competent vector and contributing to a better understanding of the eco-epidemiology of relapsing fever in South America.

## Introduction

Tick vector competence, defined as the ability to acquire, maintain, and transmit pathogens, is influenced by factors such as host-seeking behavior, duration of host contact, transstadial perpetuation, and transovarial transmission of the pathogen (Vial 2009; Sonenshine and Roe 2014). For transmission to be effective, the pathogen must replicate sufficiently within the vertebrate host to reach a burden that enables uptake by the vector. Additionally, rapid adaptation to the vector’s internal environment, which differs physiologically and immunologically from that of the vertebrate host, is essential for the pathogen’s survival and perpetuation (Boyle et al. 2014; Estrada-Peña et al. 2018; Lopez et al. 2021). Transstadial perpetuation and transovarial transmission are critical mechanisms for the maintenance of microorganisms within vector populations across multiple generations, representing key strategies employed by various pathogens. Therefore, understanding the interactions among the mammalian host, the pathogen, and the vector is essential for the development of effective strategies to control vector-borne diseases (Neelakanta et al. 2016; Embers et al. 2019; Lopez et al. 2021).

Tick-borne relapsing fever is a disease caused by bacterial agents of the genus *Borrelia* phylogenetically assigned to the relapsing fever group (RFG). Except for one species (*Borrelia recurrentis*), which is transmitted by the human body lice, all RFG *Borrelia* species are transmitted by ticks (Trevisan et al. 2021). Although there are a few *Borrelia* species associated with hard ticks (Ixodidae family), most of the tick-borne RFG borreliae are associated with soft ticks (Argasidae family), chiefly of the genus *Ornithodoros* (Trevisan et al. 2021).

Most of the tick-borne RFG borreliae are zoonotic agents that circulate between vertebrate animals and soft ticks in different regions of the world, causing human disease (relapsing fever) when infected ticks bite humans (Cutler 2015). Despite the widespread occurrence of tick-borne RFG borreliosis in temperate and tropical regions of the world (Talagrand-Reboul et al. 2018; Trevisan et al. 2021), the disease has been notably neglected in South America (Faccini-Martínez et al. 2022). Human cases of tick-borne RFG borrelial infectione have been reported in Colombia and Venezuela, with all the reports restricted to the first half of the 20th century. These South American cases, together with others reported in Panama during the same period, were attributed to the RFG agent *Borrelia venezuelensis* transmitted by its vector tick *Ornithodoros rudis* (Faccini-Martínez et al. 2022).

It was only recently that *O. rudis* was confirmed in Brazil; previous reports cast doubt on its presence in the country (Dantas-Torres et al. 2009). During field work in 2017, Muñoz-Leal et al. (2018) collected 30 specimens of *O. rudis* (25 adults and 5 nymphs) from debris of a bird nest inside a hollow palm-tree at Riachão Municipality, Maranhão State, northeastern Brazil. Attempting to isolate tick-borne spirochetes, the authors fed the adult ticks (10 males, 15 females) on naïve vesper mice (*Callomys callosus*). One animal, upon which a female *O. rudis* was fed, showed motile spirochetes in blood using dark-field microscopy (Muñoz-Leal et al. 2018). The spirochetemic blood was further processed by in vitro isolation (Muñoz-Leal et al. 2018); the genome of the borrelial isolate was published as *B. venezuelensis* (Kneubehl et al. 2022).

The incrimination of *O. rudis* as a vector of *B. venezuelensis* was based on studies carried out in Colombia, Panama and Venezuela, in which spirochetemia was demonstrated in laboratory mammals (mice, rats) and in a human, after inoculation with field-collected body homogenates of *O. rudis* (Dunn 1927; Dunn and Clark 1933; Pifano et al. 1941). In one of these studies (Dunn and Clark 1933), vector competence was demonstrated by exposing a human volunteer to infestations by several stages of *O. rudis* ticks that had been collected from a household where human cases of relapsing fever had been reported. In addition, *O. rudis* first instar-nymphs (N1) transmitted *B. venezuelensis* to a monkey (*Macaccus rhesus*) after larval-acquisition feeding on a spirochetemic rat, demonstrating transstadial perpetuation of the agent from larva to N1 nymph, and the vector competence of the latter (Dunn and Clark 1933). More recently, vector competence was demonstrated for a field-collected *O. rudis* female from Brazil, which transmitted *B. venezuelensis* to a vesper mouse in the laboratory (Muñoz-Leal et al. 2018). To our knowledge, no further studies on vector competence or transstadial perpetuation have been reported for *B. venezuelensis*, for which the transovarial transmission remains untested.

In the present study, we used the colony of *O. rudis* from the study of Muñoz-Leal et al. (2018) to evaluate transovarial and transstadial perpetuation of *B. venezuelensis*, and the vector competence of larvae, nymphs, and adults of *O. rudis* from Brazil.

### Materials and methods

In the study of Muñoz-Leal et al. (2018), field-collected *O. rudis* adults (15 females and 10 males) were allowed to feed on tick-naïve vesper mice. After feeding, some of the 15 females (generation F_0_) produced offspring, which were all pooled and used to establish a laboratory colony. The F_1_ offspring were reared to F_1_ adults by feeding on tick-naïve hamsters (*Mesocricetus auratus*), which were not tested for borrelial infection after the feeding of each tick stage. After this initial procedure, the present study started with 56 F_1_ adult ticks (22 males, 34 females).

**Fig. 1:**
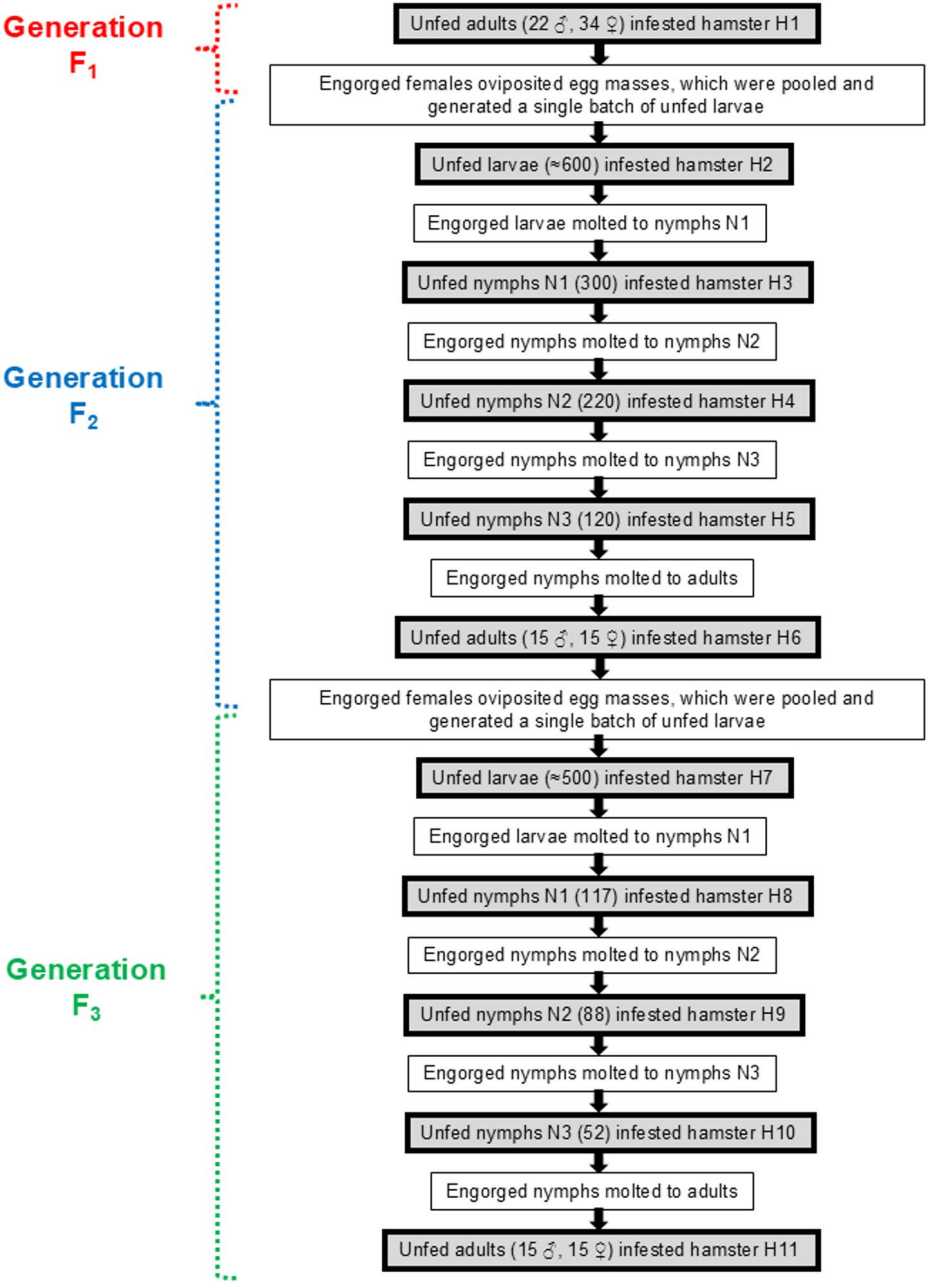
Diagram illustrating experimental procedures with the laboratory colony of *Ornithodoros rudis* ticks from F_1_ adults to F_3_ adults. Each tick stage/instar from one generation fed on a single hamster (numbered from H1 to H11). Tick off-host development (molting, oviposition and egg incubation) occurred in an incubator at 26 °C and 80% relative humidity

The 56 F_1_ adult ticks (22 males, 34 females) were allowed to feed to repletion on a hamster. After that, the offspring of these females were pooled (F_2_ larvae) and reared to the adult stage (F_2_ adults) by feeding the ticks of each stage (larvae, nymphs, or adults) or each nymphal instar (N1, N2 and N3) on a single hamster. Thereafter, the F_3_ offspring were reared to the adult stage by feeding F_3_ larvae, the three nymphal instars (N1, N2 and N3) and adults, each on a single hamster (Fig. 1).

For tick feeding, each hamster was chemically anesthetized using an intramuscular injection of a combination of ketamine (80 mg/kg) and xylazine (10 mg/kg). After sedation, the ventral region of each rodent was shaved to facilitate tick attachment. Ticks were placed in a plastic feeding chamber (Levin and Schumacher 2016) and allowed to feed until full engorgement (20–60 min). Upon detachment, fully engorged ticks were transferred to labeled plastic tubes (6 cm long, 4 cm wide) containing paper to absorb humidity and maintained in an incubator.

Off-host developmental stages of the ticks were maintained in an incubator under controlled conditions of 26 °C, 80% relative humidity, and 24 h-scotophase/day. All the hamsters used in this study came from a laboratory animal room, with no previous contact with ticks or tick-borne agents. Hamsters were used for feeding a single tick stage/instar and generation with no hamster used for more than one infestation. Before infestation with ticks, a drop of blood (2.5 µl, measured with a micropipette) was obtained from each hamster by ear vein-puncture, expressed onto glass slides, and visually monitored by dark-field microscopy to detect the presence of motile spirochetes, as previously described (Oliveira et al. 2023). This procedure was repeated daily up to 21 days after infestation (DAI). When detected, the total number of spirochetes per sampling day was calculated by summing the number of motile spirochetes counted in a total of 20 microscope fields at 200× magnification.

From each infested hamster, a blood sample (50–100 µl) was collected between 12 and 15 DAI through eye capillary puncture, after chemical anesthesia as described above. DNA was extracted from hamster blood using the PureLink Genomic DNA Mini Kit (Invitrogen, Carlsbad, CA, USA), according to the manufacturer’s instructions. Hamster blood DNA was tested by a *Borrelia-*specific real-time PCR assay using primers Bor16S3F and Bor16S3R (and an internal probe Bor16S3P) targeting a 148 bp fragment of the *Borrelia* 16 S rRNA gene, as described (Parola et al. 2011). To confirm that the detected borreliae was *B. venezuelensis*, some samples positive by real-time PCR were tested by a conventional PCR assay targeting a 354-bp fragment of the borrelial *flaB* gene (Stromdahl et al. 2003). PCR products were sequenced and submitted for BLAST analysis (www.ncbi.nlm.nih.gov/blast) to determine the closest identities available in GenBank. In all PCR assays, *Borrelia anserina* DNA and water were used as positive and negative controls, respectively.

This study was approved by the Institutional Animal Care and Use Committee (IACUC) of the Faculty of Veterinary Medicine of the University of São Paulo (protocol number 2458021221).

### Results

A total of 11 hamsters (numbered from H1 to H11) were used, one for feeding each tick stage/instar from one generation, from F_1_ adults to F_3_ adults (Table 1). During the 21-day course of dark-field microscopy monitoring of hamsters, motile spirochetes were visualized in blood several days after tick infestations on hamsters H1, H2, H5, H6, and H7, as detailed in Table 1. Hamster H3 and H4 died at 5 and 7 DAI, respectively, without spirochetes been observed in blood. These two hamsters developed neurological disorders (ataxia, tremors) and died suddenly with generalized paralysis. The spleen of hamster H3 was collected and tested by the real-time PCR assay, but no *Borrelia* DNA was detected. Hamster H4 was not molecularly tested.

**Table 1:** Data on infestations of 11 hamsters (H1 to H11) with different stages of a laboratory colony of *Ornithodoros rudis* that was previously shown to be naturally infected with *Borrelia venezuelensis.* Each infested hamster has its blood daily examined for the presence of motile spirochetes by dark field microscopy from 0 to 21 days after infestation (DAI)

Hamster no.	Tick Generation	Tick stage/instar	No. ticks that fed on the hamster	DAI that motile spirochetes were visualized in hamster blood
H1	F_1_	Adults	56	5 to 14
H2	F_2_	Larvae	≈ 600	7 to 13
H3	F_2_	Nymphs N1	300	none*
H4	F_2_	Nymphs N2	220	none*
H5	F_2_	Nymphs N3	120	10 to 16
H6	F_2_	Adults	30	11, 13 to 17
H7	F_3_	Larvae	≈ 600	14 to 18
H8	F_3_	Nymphs N1	117	none
H9	F_3_	Nymphs N2	88	none
H10	F_3_	Nymphs N3	52	none
H11	F_3_	Adults	30	none

*Hamsters H3 and H4 died at 5 and 7, respectively, days after infestation with ticks

Transmission of borreliae was observed in hamster H1 (infested with F_1_ adults), indicating that the *O. rudis* colony remained infected by *B. venezuelensis* from the F_0_ adult female reported by Muñoz-Leal et al. (2018). Thereafter, F_2_ larvae successfully transmitted borreliae to hamster H2, indicating that *B. venezuelensis* was transmitted transovarially from F_1_ females to F_2_ larvae. Transmissions of *B. venezuelensis* by N1 and N2 nymphs (F_2_) could not be demonstrated because the two infested hamsters (H3 and H4) died (Table 1). The subsequent stage, N3, successfully transmitted borreliae to hamster H5, as did the F_2_ adult ticks to hamster H6. The F_3_ larvae successfully transmitted borreliae to hamster H7, indicating that *B. venezuelensis* was transmitted transovarially from F_2_ females to F_3_ larvae. However, borrelial transmission to hamsters could not be demonstrated in any of the subsequent stages/instars of F_3_ ticks (nymphs N1 to adults feeding on hamsters H8 to H11) (Table 1).

**Fig. 2:**
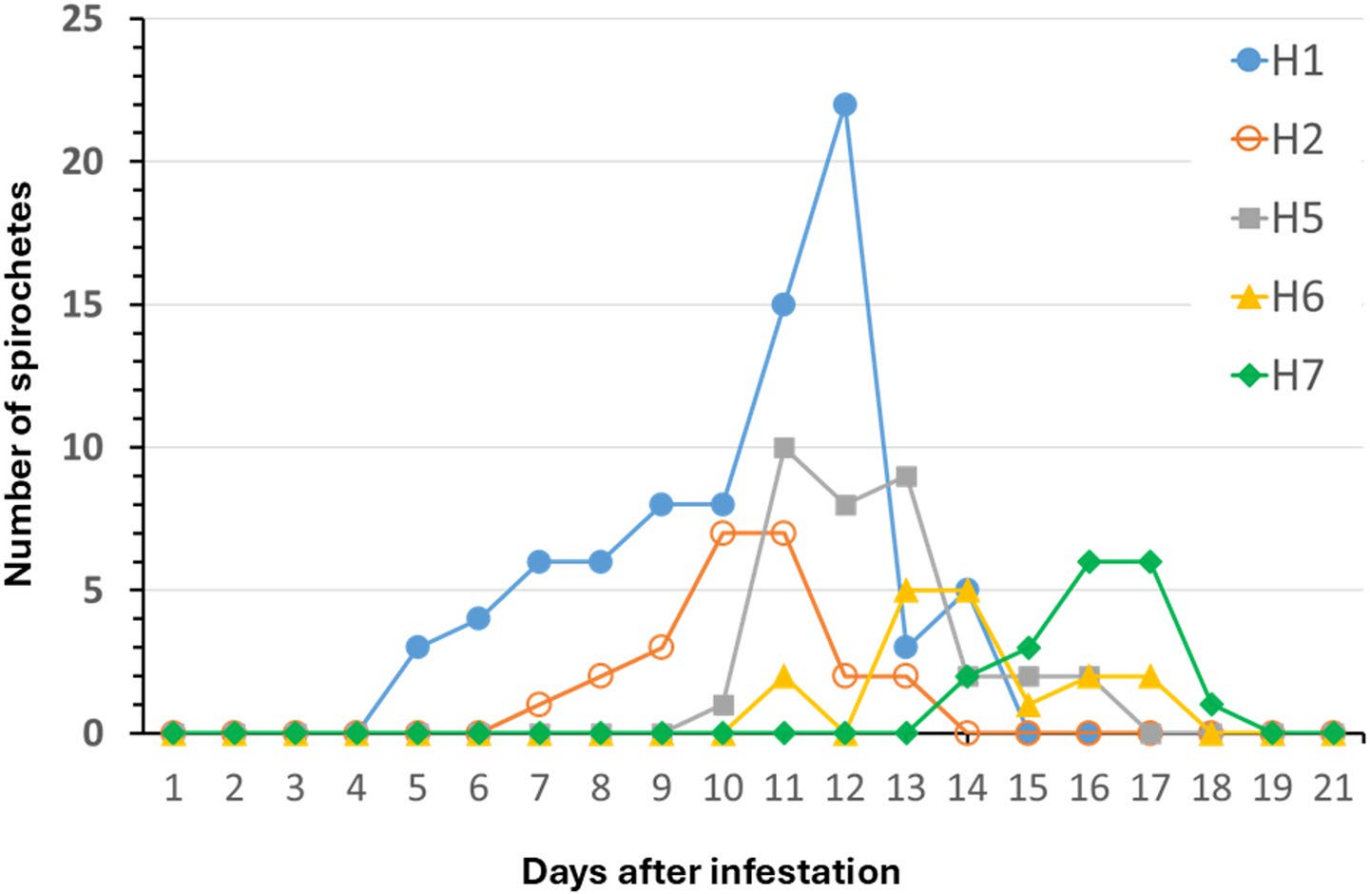
Results of dark-field examination of blood samples of hamsters H1, H2, H5, H6 and H7 on days after infestation with *Ornithodoros rudis*. Values represent the total number of motile spirochetes per 20 microscope fields at 200x magnification on each sampled day

When motile spirochetes were detected in hamster blood, the number of spirochetes counted in a total of 20 microscopic fields varied from 1 to 22 per day, with maximum counts at 10 to 12 DAI for hamsters H1, H2 and H5, 13–14 DAI for H6, and 16–17 DAI for H7 (Fig. 2). Borrelial DNA was detected in hamster blood by real-time PCR at 12 DPI in hamsters H1 and H2, and at 15 DPI in hamsters H5, H6 and H7. No borrelial DNA was detected in the blood collected at 15 DPI from hamsters H8 to H11. Blood from hamsters H6 and H7 were tested by conventional PCR and sequencing, which generated a *flaB* partial sequence that was 100% identical (300/300 bp, after removal of primer sequences from the flanks) to the GenBank *flaB* sequence of *B. venezuelensis* (MG651650).

### Discussion

This study provides experimental demonstration of transstadial perpetuation of *B. venezuelensis* among all feeding stages of *O. rudis* for three generations. Previous studies with *O. rudis* demonstrated limited transstadial perpetuation, from larva to the first nymphal instar only (Dunn and Clark 1933). Here we demonstrated transovarial transmission of *B. venezuelensis* during two consecutive generations of *O. rudis.* Although transovarial transmission of RFG borreliae may be a common feature in *Ornithodoros* spp. (Schwan and Raffel 2021), results have varied. For instance, it was successfully demonstrated for *Borrelia crocidurae* in *Ornithodoros erraticus* but not in *Ornithodoros savignyi* (Gaber et al. 1984), whereas transovarial transmission of *Borrelia duttonii* in *Ornithodoros moubata* varied among different borrelial strains (Tabuchi et al. 2008). Successful transovarial transmission in the present study might be related to using a population of *O. rudis* that was naturally infected with *B. venezuelensis.*

Vector competence, as determined by the ability to transmit *B. venezuelensis*, was demonstrated for the three feeding stages (larvae, nymphs and adults) of *O. rudis.* These findings complement previous studies that demonstrated transmission competence for N1 nymphs (Dunn and Clark 1933), a female tick (Muñoz-Leal et al. 2018) and pooled feeding stages (Dunn and Clark 1933). Surprisingly, after transmission and maintenance of the borrelial infection throughout all tick developmental stages from F_1_ adults to F_3_ larvae, transmission of *B. venezuelensis* was not demonstrated for F_3_ post-larval stages. This result could be related to low infection rates by *B. venezuelensis* in F_1_ and F_2_ ticks. Because the infestations with F_3_ nymphs consisted of much lower number of individuals than in the infestations with F_2_ nymphs, it is possible that the infection by *B. venezuelensis* was lost in F_3_ post-larval stages. This statement is based on the possible low infection rate of the field collected adults, for which Muñoz-Leal et al. (2018) demonstrated transmission of *B. venezuelensis* to mice for only one (4%) out of 25 field-collected adults. Alternatively, it is possible that the F_3_ nymphs still harboured a very low load of spirochaetes, which was insufficient to cause infection in their hosts. Unfortunately, these ticks were not molecularly tested to confirm this possibility.

The two hamsters that died of paralysis (H3 and H4) were precisely those that were infested with a greater number of nymphs, three to five times higher than the numbers of nymphs used in the other hamsters in this study. The absence of borrelial DNA amplification in the blood and organs of one of the hamsters indicates that the death was not related to infection with *B. venezuelensis*. In a study with *Ornithodoros* aff. *puertoricensis*, Venzal et al. (2007) observed that mice exposed to higher tick loads showed nervous incoordination (ataxia and tremors) from 5 DAI, dying by the 8th DAI. They ruled out the role of any tick-borne pathogen and concluded that the animals died of tick toxicosis. Since the clinical picture presented by hamsters H3 and H4 was similar, it is possible that they also died of tick toxicosis due to high tick load.

From an ecological and epidemiological perspective, the results of this study reinforce the relevance of *O. rudis* as a competent vector of *B. venezuelensis* and suggest that this tick species may also act as a reservoir for the bacterium in natural environments, contributing to its persistence even in the absence of infected vertebrate hosts. This is particularly relevant in settings such as caves, nests, or shelters of wild animals, where argasid tick density can be high and human contact is occasional, yet sufficient to trigger sporadic outbreaks of relapsing fever. Moreover, this study provides a solid experimental foundation for future research aimed at developing surveillance, control, and prevention strategies for *Borrelia*-associated relapsing fever in Brazil. The confirmation of vector competence and the ability of *O. rudis* to maintain the pathogen may support public health policies grounded in the One Health approach, integrating veterinary, environmental, and human health actions.

## Conclusions

This study demonstrated, for the first time, the transstadial perpetuation among all tick developmental stages and transovarial transmission of *B. venezuelensis* in *O. rudis*. The infection was maintained across multiple tick developmental stages and transmission to vertebrate hosts, providing strong evidence of vector competence. These findings underscore the importance of argasid ticks in the maintenance of pathogenic *Borrelia* species and expand our understanding of the ecology of tick-borne diseases.

## Data Availability

We declare all data is being provided within this manuscript.
